# P-Cadherin Linking Breast Cancer Stem Cells and Invasion: A Promising Marker to Identify an “Intermediate/Metastable” EMT State

**DOI:** 10.3389/fonc.2014.00371

**Published:** 2015-01-05

**Authors:** Ana Sofia Ribeiro, Joana Paredes

**Affiliations:** ^1^Institute of Molecular Pathology and Immunology of the University of Porto (IPATIMUP), Porto, Portugal; ^2^Department of Pathology and Oncology, Faculty of Medicine of the University of Porto, Porto, Portugal

**Keywords:** P-cadherin, EMT transition, breast cancer, metastasis, metastable phenotype

## Abstract

Epithelial–mesenchymal transition (also known as EMT) is a fundamental mechanism occurring during embryonic development and tissue differentiation, being also crucial for cancer progression. Actually, the EMT program contributes to the dissemination of cancer cells from solid tumors and to the formation of micro-metastasis that subsequently develop into clinically detectable metastases. Besides being a process that is defined by the progressive loss of epithelial cell characteristics and the acquisition of mesenchymal features, EMT has also been implicated in therapy resistance, immune escape, and maintenance of cancer stem cell properties, such as self-renewal capacity. However, the majority of the studies usually neglect the progressive alterations occurring during intermediate EMT states, which imply a range of phenotypic cellular heterogeneity that can potentially generate more metastable and plastic tumor cells. In fact, few studies have tried to identify these transitory states, partly due to the current lack of a detailed understanding of EMT, as well as of reliable readouts for its progression. Herein, a brief review of evidences is presented, showing that P-cadherin expression, which has been already identified as a breast cancer stem cell marker and invasive promoter, is probably able to identify an intermediate EMT state associated with a metastable phenotype. This hypothesis is based on our own work, as well as on the results described by others, which suggest the use of P-cadherin as a promising EMT marker, clearly functioning as an important clinical prognostic factor and putative therapeutic target in breast carcinogenesis.

## EMT: Epithelial to Mesenchymal Transition

Epithelial–mesenchymal transition (EMT) is a highly regulated transdifferentiation cellular program, by which static and polarized epithelial cells convert to an invasive and motile mesenchymal morphology. During the EMT process, there is the progressive loss of epithelial characteristics and the acquisition of mesenchymal features, which, in a cancer context, leads to the development of cells that are chemoresistant, able to escape to immune cells and with stem cell properties. Moreover, this phenotypic transformation gives cancer cells the ability to invade locally, to resist apoptosis, and to metastasize (1–4). Interestingly, the phenotypic plasticity afforded by EMT is revealed by the occurrence of the reverse process, a mesenchymal–epithelial transition (or MET) (5–7), which involves the conversion of mesenchymal cells to an epithelial cell phenotype.

### EMT states

Epithelial–mesenchymal transition is a multistep program that involves a series of changes by which epithelial cells lose their epithelial characteristics and acquire properties that are typical of mesenchymal cells. One of the earliest events during EMT involves the decrease in proteins from tight and adherent junctions (8). Epithelial-like cells exhibit an organized apical–basal polarity maintained by the precise arrangement of actin filaments and adhesive structures, such as tight junctions, adherens junctions, and desmosomes (9). Specialized adhesive molecules, such as cadherins, integrins, and other cell-surface proteins, are essential for the maintenance of the epithelial phenotype by stabilizing cell–cell contacts. Thus, a decreased expression of these proteins complex triggers redistribution of key molecules at the cell surface, disruption of the polarity complex, and cytoskeletal reorganization (10). Following loss of junctional complexes and downregulation of E-Cadherin (epithelial cadherin), β−catenin is no longer sequestered in the cytoplasm and translocates to the nucleus to activate β-catenin responsive genes. In the nucleus, a transcriptional shift is observed, where several transcription factors suppress epithelial markers and activate mesenchymal genes, promoting a mesenchymal-like phenotype (11). At this stage, cytoskeletal proteins are upregulated, as is the deposition of the extracellular matrix (ECM) components. All these alterations stimulate integrin signaling and promote the formation of focal adhesion complexes, leading in this way to an increase in cell motility and invasion capacity.

Conversely to epithelial cell phenotype, mesenchymal cells are characterized by a unique spindle morphology defined by a front-back-end polarity and enhanced invasive potential (12, 13). However, not all cells undergo a complete EMT; instead, most cells undergo partial EMT, an intermediate state in which cells retain some cell–cell adhesion characteristic of epithelial cells, but gain some migratory ability, that is a feature of mesenchymal cells (1). This means that cancer cells in this transient EMT phenotype are endowed with special properties, such as collective cell migration and invasion. Moreover, the transient phenotypic changes during EMT have been also associated with the acquisition of stem-like properties. Actually, the intermediate EMT stage has been coined as the metastable phenotype (14), since it describes the simultaneous existence of both epithelial and mesenchymal characteristics, being of great importance for the understanding of the major cellular changes associated with the progression of the EMT program.

Curiously, the identification of EMT transient stages has been neglected, although it is thought to be more prevalent than the pure mesenchymal cell morphology. In fact, it has been accepted by several authors that this hybrid phenotype, reflecting epithelial–mesenchymal plasticity, should be included in these intermediate states of EMT, implying a range of phenotypic cellular heterogeneity that can generate more plastic and metastable tumor cells (1). Interestingly, the metastatic capacity of these cells is consistent with the expression of stem cell markers in colorectal cells undergoing EMT (15), suggesting that such plasticity may be found in progenitor cells in various organs. This plasticity can explain why it is so rare to observe cells undergoing EMT during cancer progression, and also that the acquisition of mesenchymal characteristics may be transitory, undergoing a reverse process during the last stages of tumorigenesis (6).

Due to the tremendous difficulty in capturing cells in the intermediate states of EMT (16), most studies have been focused in finding biomarkers that identify the non-invasive full epithelial state, as well as the aggressive, motile, and invasive mesenchymal state.

### Classical EMT markers

The conversion of epithelial-like cells into mesenchymal-like cells requires alterations in cellular morphology, adhesion, and migratory capacity. The different degrees of EMT can occur in carcinomas, being the alterations found in the expression of molecular markers used to assess the EMT status (17).

At the molecular level, the EMT transition is characterized by a series of coordinated changes including downregulation of epithelial markers (e.g., cytokeratin 8, 18, 19, E-cadherin, claudins, occludins) and upregulation of mesenchymal markers (e.g., vimentin, N-cadherin) (18), what results in numerous phenotypic changes, such as the loss of cell–cell adhesion and cell polarity, and the acquisition of migratory and invasive properties (3, 15). The cell intermediate filament status changes from a keratin-rich network, which connects to adherens junctions and hemidesmosomes, to a vimentin-rich network connecting to focal adhesions.

The loss of functional E-cadherin containing junctions with the concomitant upregulation of N-cadherin (19, 20) is termed cadherin switch and is a hallmark of the EMT process. This switch has been already reported for carcinomas in the esophagus, prostate, cervix, and ovary (20–23), being associated with tumor progression and metastatic disease.

Epithelial–mesenchymal transition process is also particularly associated with the expression of zinc-finger transcription factors Snail (SNAI1) and Slug (SNAI2), as well as of ZEB1 (zinc-finger E-box-binding homeobox 1), ZEB2, FoxC2 (forkhead box protein C2), and TWIST (24, 25). Moreover, several evidences show that the expression of the intermediate filament protein vimentin can be upregulated by different EMT transcription factors in a direct or indirect way (26–30). Both mesenchymal proteins, vimentin and fibronectin, contribute to changes in cytoskeletal architecture and migratory potential of cancer cells (27). All these alterations cause cells to change to a mesenchymal morphology and to a functional change toward migration, invasion, and resistance to apoptosis.

Concerning the transient/metastable phenotype, few specific markers have been proposed, due to the lack of studies evaluating the progressive and transitory states of EMT, as well as of reliable readouts for its progression. Knowing that, there is the need to find markers to identify this metastable phenotype, which will be crucial to understand how to clinically prevent metastasis and to give mechanistic insights to be translated into therapeutic opportunities.

## P-Cadherin: A Promising EMT Marker

Cell–cell adhesion, mediated by E-cadherin at adherens junctions, is essential for the maintenance of epithelial tissue architecture and homeostasis. Altered expression and/or function of E-cadherin play a major role in the acquisition of cell invasive properties and in the induction of EMT, as well as tumor progression. During EMT, epithelial adherens junctions are dynamically regulated, which trigger signaling pathways and alterations in the organization of the actin cytoskeleton that are involved in the induction of cell motility. In fact, the well-known cadherins switch, namely the loss of E-cadherin and the gain of N-cadherin, is described as being characteristic of EMT and is associated with tumor cell invasion. However, there are examples of highly aggressive and invasive tumors, like basal-like breast carcinomas, where E-cadherin expression is rarely lost, and N-cadherin is hardly overexpressed (31). In contrast, these tumors usually overexpress other classical cadherin, named P-cadherin, mainly in a wild-type E-cadherin context, which intriguingly has never been linked to the EMT process. However, since it has been already proven that P-cadherin is able to interfere with epithelial cell–cell adhesion and to promote cancer cell invasion and metastasis (32), it is our belief that P-cadherin can be also used as a new EMT marker, mainly to identify an intermediate and transient EMT state associated with a metastable phenotype.

### P-cadherin is a factor of poor prognosis in breast cancer

The role of P-cadherin in breast carcinogenesis has been extensively studied in the last few years. Presently, it is known that this protein is *de novo* expressed in near 30–40% of invasive breast carcinomas, being reported as a valuable prognostic factor in this disease. P-cadherin-positive carcinomas are significantly associated with tumors of high histological grade, with short-term overall and disease-free survival, as well as with distant and loco-regional relapse-free interval (31, 33–35). P-cadherin expression still shows a strong correlation with invasion of the vascular and soft tissues (36). Moreover, the overexpression of this protein has also been positively associated with well-established markers and biological parameters associated to poor prognosis in breast cancer, such as epidermal growth factor receptor (EGFR), cytokeratin 5 (CK5), vimentin, p53 and HER2, high proliferation rates (MIB-1), mitotic index, and decreased cell differentiation (31, 33, 34, 37). P-cadherin expression was also inversely related with age at diagnosis, hormonal receptors (ER and PgR) and Bcl-2 expression (31, 33–35).

Interestingly, P-cadherin is a marker of triple-negative (which means negative for ER, PgR, and HER2) basal-like breast carcinomas, which comprise a heterogeneous group of tumors that accounts for up to 15% of all breast cancer cases (31). These tumors are highly aggressive, affect younger patients, are more prevalent in African-American women, and often are present as interval cancers. Histologically, the majority of basal-like breast cancers are IDC-NST (invasive ductal carcinomas of no special type), high histological grade, and characterized by exceptionally high mitotic indices, the presence of central necrotic or fibrotic zones, pushing borders, conspicuous lymphocytic infiltrate, and typical/atypical medullary features (38). As the name indicates, these tumors express genes and proteins usually found in basal/myoepithelial cells of the normal breast, including high-molecular-weight cytokeratins (5/6, 14, and 17), P-cadherin, caveolins 1 and 2, nestin, αB crystallin, CD109, and EGFR (39). Since, until today, these tumors do not harbor any therapeutic target usually used to treat breast cancer patients, all these proteins, including P-cadherin, can be putative therapeutic options to be targeted.

### P-cadherin and cancer cell invasion

**Figure 1:**
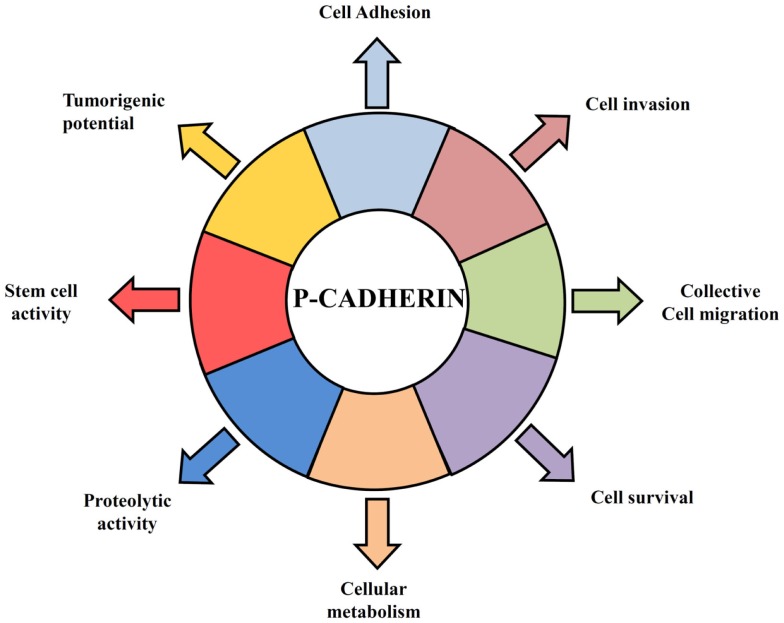
**Hallmarks of P-cadherin function in breast cancer cells**. P-cadherin overexpressing cells acquire features that give them an advantage to survive in a hostile environment leading to an invasive and tumorigenic phenotype of breast cancer cells. P-cadherin expression affects cell–cell adhesion, since it disrupts the normal suppressor function of E-cadherin, by decreasing the interaction between E-cadherin and intracellular catenins. Overexpression of this protein in breast cancer cells promotes an increase in cell migration and cell invasion, being able to provoke the secretion of pro-invasive factors, such as MMP1 and MMP2, which then lead to P-cadherin ectodomain cleavage (sP-cad) that also has pro-invasive activity by itself. Moreover, P-cadherin expression mediates cancer stem cell properties, conferring resistance to x-ray-induced cell death and being related with a hypoxic, glycolytic, and acid-resistant phenotype in breast cancer cells.

In contrast to E-cadherin, which is an important invasion suppressor protein, it has been shown that P-cadherin behaves as an invasion promoter in several cancer models, including breast cancer. It has been often reported that P-cadherin induces increased tumor cell motility and invasiveness when aberrantly overexpressed (40–46). Our group has demonstrated that one of the mechanisms underlying the invasive capacity of P-cadherin overexpression in breast cancer cells is mediated by the secretion of matrix metalloproteases (MMPs), which degrade the ECM during invasion (44, 45) and cleave P-cadherin extracellular domain to produce a soluble P-cadherin fragment (sP-cad). Interestingly, we have demonstrated that this fragment has pro-invasive effects in non-invasive breast cancer cells (45) (Figure 1). Accordingly, it was demonstrated a significant increased shedding of sP-cad in nipple aspirate fluids from women with breast cancer, when compared with healthy subjects or with women with pre-cancer conditions, suggesting its release via proteolytic processing in cancer cells (47).

Furthermore, the invasive phenotype mediated by P-cadherin was seemingly dependent on the concomitant expression of wild-type E-cadherin: in cell models where P-cadherin showed an invasion promoter function, E-cadherin was also expressed (44, 46, 48, 49); contrarily, in models, which only express P-cadherin, this protein was described as an invasion suppressor (50–52). This dual functional role of P-cadherin was recently explained by the induction of an aggressive biological cell behavior in cells co-expressing both cadherins compared to cells just expressing one of each cadherin. Indeed, we have found that P-cadherin expression disrupts the normal invasive suppressor function of E-cadherin (32) by destabilizing the normal cadherin/catenin complex (53). The induced-delocalization of β-catenin and p120-catenin from the membrane to the cytoplasm alters the actin cytoskeleton polymerization, and promotes cell migration and motility, as well as an increased invasive and tumorigenic potential (32, 46) (Figure 1).

### P-cadherin as a cancer stem cell marker

Breast carcinomas, as well as other solid tumors, contain a small population of cancer cells that have the ability to self-renew and to be tumorigenic, generating all the distinct cancer cells present within the tumor. These cells were called breast cancer stem cells (CSCs), which are the apex of a hierarchy that is comparable to the one established in normal tissues (54). Breast CSCs share a large amount of properties with mammary stem cells, namely the ability to resist to standard cancer therapies, such as radiation and chemotherapy, allowing them to survive and to cause tumor recurrence and metastasis (55–58). Targeting breast CSCs, in combination with current therapies, is the forthcoming goal in breast cancer treatment.

In this context, we have demonstrated that P-cadherin expression is able to identify basal-like breast cancer cells with stem cell properties. Using breast cancer cell lines and primary tumors, we showed that P-cadherin was directly associated with the expression of the breast stem markers CD44, CD49f, and aldehyde dehydrogenase 1 (ALDH1) in the basal-like molecular subtype. Moreover, P-cadherin-enriched cancer cell populations comprised increased *in vitro* mammosphere-forming efficiency, as well as increased tumorigenicity. Additionally, the expression of this adhesion molecule still conferred resistance to x-ray-induced cell death, sustaining a role for this molecule in another stem cell property (59) (Figure 1).

Importantly, we found that P-cadherin expression was associated with stem-/progenitor-like phenotypes of the breast, including the luminal progenitor population, CD49f^+^CD24^+^. Accordingly, Nassour et al. recently demonstrated that the expression of Slug, P-cadherin, and CD49f has a role in the growth dynamics of a subpopulation of cycling progenitor basal cap and duct cells during mammary morphogenesis (60). Moreover, we were able to establish that there is a crosstalk between the expression of P-cadherin and CD49f (α6 integrin). Actually, we have demonstrated that P-cadherin regulates the laminin receptor α6β4 integrin-signaling pathway, which activation explains the stem cell and invasive properties induced by P-cadherin to breast cancer cells (61).

Cancer stem cells are also usually described as hypoxia-resistant, presenting a preponderant glycolytic metabolism. These characteristics are also found enriched in basal-like breast carcinomas, which show increased expression of cancer stem cell markers (62). Interestingly, we demonstrated that cancer cell populations harboring high levels of P-cadherin were the same exhibiting more GLUT1 and CAIX expression. Moreover, its silencing significantly decreased the mammosphere-forming efficiency in the same range as the silencing of HIF-1α, CAIX, or GLUT1, substantiating that all these markers are being expressed by the same breast cancer stem cell population (63) (Figure 1).

### P-cadherin as a potential therapeutic target

All the knowledge that was acquired concerning P-cadherin expression in cancer supported the development of anti-P-cadherin therapeutic strategies. A humanized monoclonal antibody (PF-03732010) was developed to antagonize P-cadherin-regulated cell–cell adhesion and the associated signaling pathway. Actually, a study using this antibody confirmed the role of P-cadherin as a molecule involved in cell invasion, as well as in metastization. The authors observed that PF-03732010 treated cells and tumors showed disrupted P-cadherin signaling and resulted in anti-tumorigenic and anti-metastatic activity (64).

**Figure 2:**
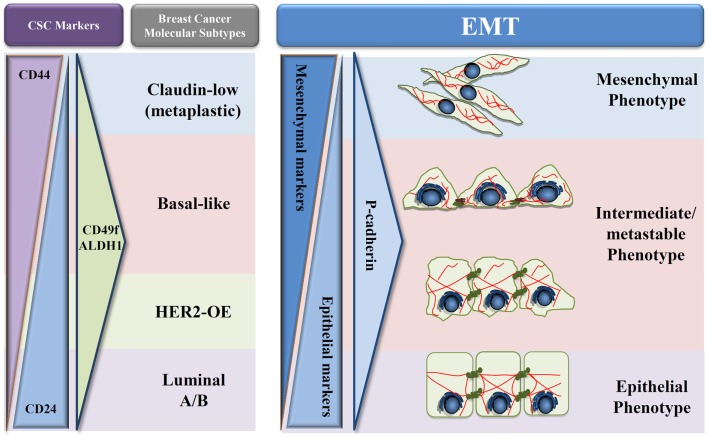
**Proposed model of P-cadherin expression in EMT progression**. Schematic representation adapted from Schmitt et al. (54) of the different types of breast cancer in what concerns cancer stem cells proteins (CD24, CD44, CD49f, and ALDH1), EMT markers, and P-cadherin expression during EMT progression. A decrease of epithelial proteins with a concomitant increase in mesenchymal markers is observed during the transition from an epithelial to a mesenchymal phenotype. During this process, we hypothesize that P-cadherin expression is in very low levels in both full epithelial and full mesenchymal states; however, an increased expression can be seen in the metastable and intermediate states of EMT.

Based on these results and on our own data, we hypothesize that P-cadherin could be a promising marker of the metastable and intermediate EMT phenotype (Figure 2). The rationale for this hypothesis relies on the fact that P-cadherin expression disturbs epithelial cell–cell adhesion and promotes the acquisition of a more undifferentiated cell phenotype, acquiring these cells an intermediate phenotypic state between epithelial and mesenchymal morphology. Additionally, P-cadherin overexpressing cells show increased therapy resistance, stem cell properties, and a more aggressive and invasive behavior. Finally, P-cadherin expression is a factor of poor prognosis in breast cancer, being associated to tumors with high metastatic potential. Thus, all these characteristics points to a transient EMT state, with cancer cells harboring an increased plasticity and metastability.

This premise seems to be corroborated by preliminary data that we have recently produced, where a three-protein EMT signature has been applied to a series of 500 invasive breast carcinomas, using the expression of two classical EMT markers, E-cadherin and vimentin, combined with P-cadherin expression. Our results show that P-cadherin identifies an intermediate state between the epithelial and the mesenchymal phenotypes, associating to a poor prognosis in breast cancer patients, but also with the expression of breast cancer stem cell markers. Although this data need further validation, both in independent breast cancer series and by *in vitro* and *in vivo* functional assays, our future aim is to definitely prove the crucial role of P-cadherin in identifying the EMT “intermediate/metastable” phenotype. Proving this hypothesis, we will clarify the role of P-cadherin in the EMT process, identifying it as a putative EMT marker and supporting the use of anti-P-cadherin therapeutics to metastatic breast cancer.

## Conflict of Interest Statement

The authors declare that the research was conducted in the absence of any commercial or financial relationships that could be construed as a potential conflict of interest.
